# The prevalence and risk factors of death anxiety and fear of COVID‐19 in an Iranian community: A cross‐sectional study

**DOI:** 10.1002/hsr2.706

**Published:** 2022-06-19

**Authors:** Arash Mani, Reza Fereidooni, Mohammad Salehi‐Marzijarani, Ali Ardekani, Sarvin Sasannia, Pardis Habibi, Leila Zarei, Seyed Taghi Heydari, Kamran B. Lankarani

**Affiliations:** ^1^ Research Center for Psychiatry and Behavior Science, Hafez Hospital Shiraz University of Medical Sciences Shiraz Iran; ^2^ Health Policy Research Center, Institute of Health Shiraz University of Medical Sciences Shiraz Iran

**Keywords:** anxiety, COVID‐19, death, fear, SARS‐CoV‐2

## Abstract

**Background and Aims:**

COVID‐19 has adversely impacted the public's mental health. One of the causes of psychopathology during the present pandemic is death anxiety and fear of COVID‐19. The present study aimed to determine the prevalence and risk factors of death anxiety and fear of COVID‐19 in Shiraz city, south of Iran.

**Methods:**

This cross‐sectional study was conducted among 982 participants in Shiraz from October to November 2021. Data were collected using Templer's Death Anxiety Scale and the Fear of COVID‐19 Scale. Trained interviewers collected data throughout different city districts. A data‐driven approach (latent class analysis) was applied to categorize the participants and determine the risk factors.

**Results:**

Among the participants, 507 (51.6%) were female, and 475 (48.4%) were male. The participants’ mean age was 38.26 ± 15.16 years. Based on the analysis, 259 (26.4%), 512 (52.1%), and 211 (21.5%) participants had low, moderate, and severe levels of death anxiety. Also, 393 (40.06%) and 588 (59.94%) of the participants had low and high levels of fear, respectively. Higher death anxiety was significantly associated with being female, having an associate degree, being retired, share of medical expenditure from total expenditure of more than 10%, having a history of hospital admission due to COVID‐19, history of COVID‐19 in relatives, and having fear of COVID‐19. Also, being female, expenses equal to income, history of hospital admission due to COVID‐19, death in relatives, and higher death anxiety were linked to higher levels of fear of COVID‐19.

**Conclusions:**

Death anxiety and fear of COVID‐19 are closely associated with each other and affected by various sociodemographic and economic factors. Given this pandemic's unpredictable nature and chronicity, interventions at the community level to support high‐risk groups are crucial.

## 1 INTRODUCTION

Death anxiety is as old as humanity. It is defined as a psychological state arising from one's fear of death or being harmed.^1^, ^2^ Encountering situations that lead to anticipation or awareness of dying is known as death anxiety.^3^ Higher death anxiety is shown to be able to predict both the existence and severity of mental diseases.^4^, ^5^

Death anxiety has been a subject of research among different population groups. In studies among cancer patients and older adults, death anxiety was reported to be moderate to high, and higher death anxiety was associated with lower quality of life.^6^, ^7^ Determinants of death anxiety are also varied among different groups since individuals' ideas about death are influenced by a variety of demographic, social, cultural, psychological, and health aspects.^8^

With the emergence of the coronavirus disease 2019 (COVID‐19) pandemic, encountering death either in relatives or acquaintances, misleading information, and uncertain reports about the disease lead to numerous psychological consequences, such as fear and death anxiety.^9^, ^10^

Previous studies assessed the overall impact of the pandemics on mental health, while fear of death has yet to be understood entirely.^11^ Vindegaard and Benrose, in a systematic review, indicated that various sociodemographic factors, current or past medical history, psychological and social factors, and job‐related factors were correlated with symptoms of psychiatric diseases at the time of the pandemic.^12^ Lee et al. stated that much of the psychological distress, such as death anxiety during the pandemic, could be attributed to COVID‐19‐related factors.^13^ A previous study showed that death anxiety in the time of pandemic is relatively high and associated with the death of a family member from COVID‐19, religiosity and cultural norms, perceived level of stress, attitude toward COVID‐19, subjective proximity to death, coping strategies, history of close contact with COVID‐19 patients, mental illness, alcohol consumption, loneliness, perceived risk, and strategies for coping with stress.^14^

It has been almost 2 years since the beginning of the COVID‐19 pandemic, but our knowledge of death anxiety and fear of COVID‐19 goes back mainly to the early days of the pandemic. The current study varies from previous ones in that the interviews were performed in‐person, more sophisticated methods of analysis were utilized, and up‐to‐date data is provided because the public perception of COVID‐19 has evolved dramatically since the early days of the pandemic. This study aims to determine the prevalence and risk factors of death anxiety and fear of COVID‐19 in the era of the COVID‐19 pandemic.

## 2 METHODS

This cross‐sectional study was conducted among 982 participants in Shiraz, Iran, from October to November 2021. Shiraz, the capital of Fars Province, is located in the south of Iran and has a population of 1.87 million. The trend of COVID‐19 new cases and deaths in Iran during the study period can be seen in Supporting Information: Figure S1. Preventive policies adopted by the government to prevent COVID‐19 at the time of the study included mandatory face masks in public places, a night traffic ban, and closure of schools, restaurants, and cinemas.

### 2.1 Study design

Considering each of the 10 urban districts of Shiraz as a separate cluster, we applied a convenience sampling method. Participants were selected at main streets, parks, and malls in different districts and at different times. After acquiring verbal informed consent, questionnaires were completed by four trained interviewers. The interviewers used a predefined protocol to invite people to participate, including self‐introduction, asking if they lived in the current district, explaining the study, and obtaining informed consent. To minimize any selection bias, each interviewer approached each city district at least three times (morning, noon, and evening) during the study period. The inclusion criteria were individuals over the age of 18, the ability to communicate, and a desire to participate in the study. Individuals who were not residents of the district where the interview took place were excluded. The study flowchart can be seen in Supporting Information: Figure S2.

### 2.2 Data collection and instruments of measurement

First, a questionnaire consisting of demographic features, economic status, history of COVID‐19 in respondents or relatives, death from COVID‐19 in the relatives, psychiatric disorders, and suicide attempts were filled. Then, data were collected from participants using two data collection tools.

A validated Persian version of Templer's (1970) Death Anxiety Scale (DAS) was used as the psychometric tool.^15^, ^16^ It is a dichotomous questionnaire consisting of 15 statements. In the original version, six items were keyed “false,” and nine were keyed “true.” Answers conforming to the key equal a score of one.^15^ However, in the Persian version, validated by Rajabi and Bahrani in 2006, each “true” equals a score of one, and each “false” equals a score of zero.^16^ Hence, a higher DAS score indicates higher death anxiety.

To assess fear of the COVID‐19, we used the Fear of COVID‐19 Scale (FCV‐19S), developed by Ahorsu et al. in Iran.^17^ This Likert scale consists of seven items, with the score for each item ranging from one to five, and a higher score indicating greater the fear of COVID‐19.

### 2.3 Statistical analysis

A data‐driven approach was used to categorize the level of death anxiety and fear of the coronavirus. A latent class analysis (LCA) was employed to categorize participants with the same pattern of responses to questionnaires. The LCA assigns an individual to a class by examining the pattern of categorical data using probabilistic methods. The first step is to define a number of classes that are noninclusive with homogeneous participants. Then we ran LCA with the number of classes from 2 to 10. The number of extracted death anxiety and fear of COVID‐19 classes were determined by lower Bayesian Information Criterion (BIC), Akaike's Information Criterion (AIC), and clinical interpretability. The detailed information on model selection statistics for LCA is presented in Supporting Information: Figure S3. Therefore, we choose three classes for death anxiety and two classes for fear of COVID‐19 with the lowest level of BIC to ease the interpretation. Then, logistic regression was used to examine associated factors, including demographic features, economic status, COVID‐19 infection, and psychiatry disorder, with the classes. Model fitting statistics, for example, pseudo‐*R* ^2^ (Cox & Snell and Nagelkerke) and −2log‐likelihood, are provided in the results tables. Quantitative and qualitative variables were described by mean ± standard deviation (SD) and frequency distribution (percentage), respectively. Statistical package for social sciences (SPSS) version 21 and the Latent GOLD (version 5.0.0) were used to perform all statistical analyses.

## 3 RESULTS

### 3.1 Class profiles

Of the participants, 507 (51.6%) were female, and 475 (48.4%) were male. The participants’ mean age was 38.26 ± 15.16 years (range: 18–86), with 356 (36.7%), 396 (40.8%), and 219 (22.6%) in the age range of less than 30, 30–49, and equal or more than 50 years old, respectively.

Based on the LCA method with three classes, 259 (26.4%), 512 (52.1%), and 211 (21.5%) of participants had low, moderate, and severe levels of death anxiety, respectively. The class with a low level of death anxiety had a mean score of 2.01 ± 1.49 and a median of 2.00 (range = 0.00–5.00). The class with a moderate level of death anxiety had a mean score of 7.82 ± 1.93 and a median of 8.00 (range = 3.00–12.00). The mean score of the class with a high level of death anxiety was 14.10 ± 1.02, and the median of 14.00 (range = 12.00–15.00).

For fear of the COVID‐19, two groups of low and high fear were defined; 393 (40.06%) and 588 (59.94%) of the participants had low and high levels of fear, respectively. The class with the low level of fear had a mean score of 10.99 ± 2.76 and a median of 11.00 (range = 7.00–16.00), while the mean score of the group with the high level of fear was 20.90 ± 4.23, and the median was 20.00 (range = 12.00–35.00). Participants with a high level of fear of COVID‐19 had a significantly higher level of death anxiety (*p* < 0.001).

### 3.2 Logistic regression

Based on multinomial logistic regression, sex (female: odds ratio [OR] = 1.65; 95% confidence interval [CI]: 1.08–2.53), household economics status (expense lower than income: OR = 0.54; 95% CI: 0.29–0.99), share of medical expenditure from total expenditure. More than 10% (OR = 1.89; 95% CI: 1.14–3.15), COVID‐19 infection in relatives (OR = 2.79; 95% CI: 1.81–4.31), suicide attempt (OR = 0.20; 95% CI: 0.06–0.65), high levels of fear of COVID‐19 (OR = 3.08; 95% CI: 2.11–4.51) were significantly associated with moderate level of death anxiety (Table 1).

**Table 1:** Association between demographic features, economic status, COVID‐19 infection, psychiatric disorder, and fear of COVID‐19 with death anxiety based on multinomial logistic regression.

	Death anxiety			Odds ratio (95% CI) for moderate vs. low	*p* value	Odds ratio (95% CI) for high vs. low	*p* value
	Low	Moderate	High				
*Sex*							
Male	143 (30.11)	253 (53.26)	79 (16.63)	Reference	‐	Reference	‐
Female	116 (22.88)	259 (51.08)	132 (26.04)	**1.65 (1.08–2.53)**	**0.019**	1.50 (0.87–2.57)	0.139
*Age*							
<30	122 (34.27)	167 (46.91)	67 (18.82)	Reference	‐	Reference	‐
30–50	96 (24.24)	202 (51.01)	98 (24.75)	1.48 (0.85–2.60)	0.168	1.17 (0.60–2.29)	0.643
>50	39 (17.81)	135 (61.64)	45 (20.55)	1.97 (0.99–3.92)	0.054	0.94 (0.40–2.22)	0.889
*Education*							
Diploma or lower	129 (24.67)	270 (51.63)	124 (23.71)	Reference	‐	Reference	‐
Associate degree	35 (23.65)	69 (46.62)	44 (29.73)	1.28 (0.75–2.20)	0.365	**2.21 (1.20**–**4.10)**	**0.011**
Bachelor	40 (26.14)	82 (53.59)	31 (20.26)	1.60 (0.91–2.84)	0.104	1.95 (0.98–3.86)	0.056
Master or higher	55 (34.81)	91 (57.59)	12 (7.59)	1.07 (0.64–1.80)	0.798	**0.29 (0.12**–**0.68)**	**0.005**
*Job*							
Employee	136 (28.87)	248 (52.65)	87 (18.47)	Reference	‐	Reference	‐
Student	43 (29.86)	74 (51.39)	27 (18.75)	0.94 (0.53–1.66)	0.832	1.00 (0.49–2.04)	0.998
Housewife	35 (20.59)	69 (40.59)	66 (38.82)	0.55 (0.29–1.05)	0.070	1.85 (0.88–3.87)	0.104
Retired	11 (12.36)	58 (65.17)	20 (22.47)	2.01 (0.81–5.00)	0.134	**3.07 (1.03**–**9.13)**	**0.044**
Unemployed	34 (31.48)	63 (58.33)	11 (10.19)	0.78 (0.43–1.39)	0.393	**0.41 (0.18**–**0.97)**	**0.042**
*Marital status*							
Single	120 (33.24)	175 (48.48)	66 (18.28)	Reference	‐	Reference	‐
Married	129 (22.63)	306 (53.68)	135 (23.68)	1.10 (0.64–1.88)	0.734	0.94 (0.49–1.81)	0.861
Divorce or widow	10 (19.61)	31 (60.78)	10 (19.61)	1.44 (0.53–3.94)	0.478	0.77 (0.21–2.76)	0.687
*Household economics status*							
Expense more than income	92 (24.53)	194 (51.73)	89 (23.73)	Reference	‐	Reference	‐
Expense equal to income	132 (25.29)	278 (53.26)	112 (21.46)	1.03 (0.69–1.52)	0.893	0.62 (0.39–1.00)	0.051
Expense lower than income	35 (41.18)	40 (47.06)	10 (11.76)	**0.54 (0.29**–**0.99)**	**0.046**	**0.18 (0.07**–**0.47)**	**0.001**
*Share of medical expenditure from total expenditure*							
**≤**10%	227 (28.77)	402 (50.95)	160 (20.28)	Reference	‐	Reference	‐
>10%	32 (16.58)	110 (56.99)	51 (26.42)	**1.89 (1.14–3.15)**	**0.014**	1.75 (0.96–3.18)	0.067
*COVID‐19 infection in respondent*							
No	155 (30.27)	289 (56.45)	68 (13.28)	Reference	‐	Reference	‐
Without hospital admit	73 (25.8)	148 (52.3)	62 (21.91)	0.80 (0.52–1.23)	0.318	1.12 (0.65–1.91)	0.686
Hospital admit	31 (16.58)	75 (40.11)	81 (43.32)	0.62 (0.35–1.11)	0.105	**2.16 (1.14–4.09)**	**0.018**
*COVID‐19 infection in relatives*							
No	113 (39.93)	134 (47.35)	36 (12.72)	Reference	‐	Reference	‐
Yes	146 (20.89)	378 (54.08)	175 (25.04)	**2.79 (1.81–4.31)**	**0.001**	**3.73 (2.09–6.65)**	**0.001**
*Death from COVID‐19 in the relatives*							
No	196 (28.24)	342 (49.28)	156 (22.48)	Reference	‐	Reference	‐
Yes	38 (19.49)	112 (57.44)	45 (23.08)	1.09 (0.68–1.76)	0.716	0.71 (0.40–1.24)	0.223
*Psychological disorder*							
No	235 (26.2)	478 (53.29)	184 (20.51)	Reference	‐	Reference	‐
Yes	24 (28.24)	34 (40)	27 (31.76)	0.59 (0.3–1.14)	0.116	1.39 (0.66–2.93)	0.382
*Suicide attempt*							
No	246 (25.71)	505 (52.77)	206 (21.53)	Reference	‐	Reference	
Yes	13 (52)	7 (28)	5 (20)	**0.20 (0.06–0.65)**	**0.008**	**0.23 (0.06–1.00)**	**0.049**
*Fear of coronavirus disease*							
Low	160 (40.70)	179 (45.50)	54 (13.80)	Reference	‐	Reference	‐
High	99 (16.80)	332 (56.50)	157 (26.70)	**3.08 (2.11–.51)**	**0.001**	**4.01 (2.47–6.5)**	**0.001**

*Note*: The odds ratio with bold style was statistically significant; Cox and Snell pseudo *R* ^2^ = 0.26; Nagelkerke pseudo *R* ^2^ = 0.30; −2log likelihood (df) = 1426.42.

Also, education (associate degree: OR = 2.21; 95% CI: 1.20–4.10, and master degree: OR = 0.29; 95% CI: 0.12–0.68), job (retired: OR = 3.07; 95% CI: 1.03–9.13, unemployed: OR = 0.41; 95% CI: 0.18–0.97), household economic status (expense lower than income: OR = 0.18; 95% CI: 0.07–0.47), COVID‐19 infection in respondent (hospital admission: OR = 2.16; 95% CI: 1.14–4.09), COVID‐19 infection in relatives (OR = 3.73; 95% CI: 2.09–6.65), suicide attempt (OR = 0.23; 95% CI: 0.06–1.00), fear of COVID‐19 (OR = 4.01; 95% CI: 2.47–6.5) were significantly associated with high levels of death anxiety (Table 1).

Based on logistic regression, sex (OR = 3.14; 95% CI: 2.15–4.59), education (associate degree: OR = 0.41; 95% CI: 0.26–0.64, bachelor: OR = 0.24; 95% CI: 0.15–0.39, and master degree: OR = 0.37; 95% CI: 0.24–0.60), household economics status (expense equal to income: OR = 1.53; 95% CI: 1.09–2.16), COVID‐19 infection in respondent (hospital admission: OR = 1.82; 95% CI: 1.12–2.97), COVID‐19 infection in relatives (OR = 0.53; 95% CI: 0.35–0.80), death from COVID‐19 in the relatives (OR = 1.89; 95% CI: 1.26–2.83), death anxiety (moderate level: OR = 3.01; 95% CI: 2.06–4.39, high level OR = 3.78; 95% CI: 2.35–6.07) were significantly associated with fear of COVID‐19 (Table 2).

**Table 2:** Association between demographic features, economic status, COVID‐19 infection, psychiatry disorder, and death anxiety with fear of COVID‐19 based on multiple logistic regression.

	Fear of coronavirus disease		Odds ratio (95% CI)	*p* value
	Low level	High level		
*Sex*				
Male	150 (29.64)	356 (70.36)	Reference	‐
Female	243 (51.16)	232 (48.84)	**3.14 (2.15–4.59)**	**0.001**
*Age*				
<30	155 (43.66)	200 (56.34)	Reference	‐
30–50	159 (40.15)	237 (59.85)	1.22 (0.74–2.02)	0.440
>50	75 (34.25)	144 (65.75)	1.19 (0.64–2.19)	0.580
*Education*				
Diploma or lower	150 (28.68)	373 (71.32)	Reference	‐
Associate degree	63 (42.57)	85 (57.43)	**0.41 (0.26–0.64)**	**0.001**
Bachelor	89 (58.17)	64 (41.83)	**0.24 (0.15–0.39)**	**0.001**
Master or higher	91 (57.96)	66 (42.04)	**0.37 (0.23–0.60)**	**0.001**
*Job*				
Employee	218 (46.28)	253 (53.72)	Reference	‐
Student	58 (40.56)	85 (59.44)	1.26 (0.75–2.12)	0.380
Housewife	50 (29.41)	120 (70.59)	0.61 (0.34–1.08)	0.090
Retired	32 (35.96)	57 (64.04)	1.14 (0.57–2.25)	0.710
Unemployed	35 (32.41)	73 (67.59)	1.55 (0.88–2.72)	0.130
*Marital status*				
Single	153 (42.5)	207 (57.5)	Reference	‐
Married	225 (39.47)	345 (60.53)	1.09 (0.67–1.76)	0.730
Divorce or widow	15 (29.41)	36 (70.59)	1.19 (0.50–2.85)	0.690
*Household economics status*				
Expense more than income	173 (46.26)	201 (53.74)	Reference	‐
Expense equal to income	182 (34.87)	340 (65.13)	**1.53 (1.09–2.16)**	**0.020**
Expense lower than income	38 (44.71)	47 (55.29)	1.04 (0.57–1.88)	0.900
*Share of medical expenditure from total expenditure*				
≤10%	326 (41.37)	462 (58.63)	Reference	‐
>10%	67 (34.72)	126 (65.28)	1.13 (0.74–1.72)	0.580
*COVID‐19 infection in respondent*				
No	224 (43.84)	287 (56.16)	Reference	‐
Without hospital admit	117 (41.34)	166 (58.66)	1.24 (0.85–1.8)	0.270
Hospital admit	52 (27.81)	135 (72.19)	**1.82 (1.12–2.97)**	**0.020**
*COVID‐19 infection in relatives*				
No	113 (39.93)	170 (60.07)	Reference	‐
Yes	280 (40.11)	418 (59.89)	**0.53 (0.35–0.80)**	**0.001**
*Death from COVID‐19 in the relatives*				
No	287 (41.35)	407 (58.65)	Reference	‐
Yes	55(28.21)	140 (71.79)	**1.89 (1.26–2.83)**	**0.001**
*Psychological disorder*				
No	364 (40.58)	533 (59.42)	Reference	‐
Yes	29 (34.52)	55 (65.48)	1.41 (0.78–2.54)	0.250
*Suicide attempt*				
No	379 (39.64)	577 (60.36)	Reference	‐
Yes	14 (56)	11 (44)	0.38 (0.14–1.07)	0.070
*Death anxiety*				
Low	160 (61.77)	99 (38.22)	Reference	‐
Moderate	179 (35.02)	332 (64.97)	**3.01 (2.06–4.39)**	**0.001**
High	54 (25.59)	157 (74.40)	**3.78 (2.35–6.07)**	**0.001**

*Note*: The odds ratio with bold style was statistically significant; Cox and Snell pseudo *R* ^2^ = 0.20; Nagelkerke pseudo *R* ^2^ =  0.27; −2log likelihood = 974.44.

## 4 DISCUSSION

The effects of public health emergencies on mental status are well understood and reported during previous similar outbreaks, namely Ebola and severe acute respiratory syndrome (SARS).^18^, ^19^ Although previous studies mainly focused on describing the effects of the COVID‐19 pandemic on mental health, this study is one of few that scrutinize one of the contributing factors to psychopathology during the present pandemic, namely death anxiety and fear of COVID‐19, and evaluates its associated risk factors.

According to our findings, females were shown to have more death anxiety and fear of the COVID‐19. In line with our results, previous studies indicated that females are more prone to various types of psychopathology during the COVID‐19 pandemic.^20^, ^21^, ^22^ Therefore, death anxiety and fear of COVID‐19 may be responsible for a part of the current burden of mental disorders among women. Also, there was a significant association between the history of COVID‐19 in participants or their relatives and death anxiety. When an individual or their relatives get infected with the disease, they feel more threatened as their concern changes from fear of getting the disease to fear of the outcome of the disease (death or recovery).^23^ Additionally, as an infected individual seeks more information about the disease, they may encounter uncertain or inaccurate information that makes them incorrectly estimate the disease's risks.^24^

Our results showed that having an associate degree was associated with higher death anxiety. In contrast, having a master's or higher degree had a protective effect. In line with our findings, previous studies indicated that lower levels of education were associated with psychiatric symptoms.^22^, ^25^ On the other hand, some studies suggested the protective role of receiving information on COVID‐19 from scientific sources rather than social media on mental issues.^25^, ^26^ Furthermore, uncertain reports, rumors, and conspiracy theories mainly affect people with lower levels of health literacy.^27^, ^28^ Therefore, it can be hypothesized that while educated individuals seek reliable information, low educated people fail to conceptualize the disease correctly, which may predispose them to both over and underestimate the risk of the disease, sometimes resulting in exaggerated fear and anxiety.

Another way the pandemic has affected society is through its effects on employment status and the economy.^29^ Because of their low income, economically vulnerable people are more likely to worry about the treatment expenses if they get infected. This explains why economically vulnerable people had more fear of the disease.

As death anxiety and fear are intertwined with many psychological disorders, addressing them is crucial to prevent further psychopathologies. Fear of COVID‐19 in the previous studies has been linked to being female, low education, and being hospitalized, which are in line with our results.^30^, ^31^ Also, the interplay between fear of COVID‐19 and death anxiety is shown in this study, which sheds light on the necessity of interventions to improve health literacy on COVID‐19 and mitigate fear and, subsequently, death anxiety in societies.

### 4.1 Strengths and limitations

As a strength, we utilized in‐person interviews to fill out questionnaires that do not have the limitations of online surveys. Also, we took the sample from different city districts, which are representative of people with different socioeconomic statuses, educational levels, and neighborhood conditions. However, our study is not free of limitations, and the cross‐sectional design of this study prevents us from drawing firm conclusions on the causality between risk factors and variables. Also, our sample did not contain people with disabilities who cannot commute in the city. Furthermore, we acknowledge the differences in fear and death anxiety between urban and rural areas. Self‐reported nature of the study's instruments may lead to recall or social desirability bias. Also, we acknowledge potential selections bias because of the people who refused to participate in the study. Finally, our results are based on a survey in an urban area in the south of Iran; hence, the results should be interpreted with caution in terms of generalizability.

### 4.2 Implications

As fear and death anxiety are affected by a wide range of social, economic, cultural, and regional factors, further research in different parts of the world is essential to portray a clearer picture of this topic. Online photovoice (OPV), as one of the current and effective innovative qualitative research methods, can be used in future research.^32^ OPV gives opportunities to the participants to express their own experiences with as little manipulation as possible, if at all, compared to traditional quantitative methods. Also, collaborative efforts such as community‐based participatory research (CBPR) methods can be used to explore the causes and effects of the negative emotional impact of the COVID‐19 pandemic on self‐compassion and mindfulness parenting in collaboration with populations most impacted by it.^33^ Some researchers have previously employed OPV, and future researchers may be able to apply it to develop sensitive prevention and intervention programs to improve targeted populations' mental health.^34^, ^35^

## 5 CONCLUSION

Death anxiety and fear of COVID‐19 were closely associated with each other and with demographic, socioeconomic factors, and history of COVID‐19 infection. With the emergence of new variants and the unpredictable future of the present pandemic, the importance of interventions to address the root causes of the psychopathologies during the pandemic is evident.

## AUTHOR CONTRIBUTIONS

**Arash Mani**: Conceptualization; formal analysis; investigation; methodology; project administration; resources; writing—review and editing. **Reza Fereidooni**: Data curation; investigation; writing—original draft; writing—review and editing. **Mohammad Salehi‐Marzijarani**: Data curation; formal analysis; investigation; software; writing—original draft; writing—review and editing. **Ali Ardekani**: Investigation; writing—original draft; writing—review and editing. **Sarvin Sasannia**: Data curation; investigation; writing—original draft; writing—review and editing. **Pardis Habibi**: Data curation; investigation; writing—original draft; writing—review and editing. **Leila Zarei**: Data curation; investigation; writing—original draft; writing—review and editing. **Seyed T. Heydari**: Conceptualization; formal analysis; funding acquisition; methodology; project administration; resources; software; writing—review and editing. **Kamran B. Lankarani**: Conceptualization; methodology; supervision; writing—review, and editing. All authors have read and approved the manuscript.

## CONFLICT OF INTEREST

The authors declare no conflict of interest.

## TRANSPARENCY STATEMENT

The lead author affirms that this manuscript is an honest, accurate, and transparent account of the study being reported; that no important aspects of the study have been omitted; and that any discrepancies from the study as planned have been explained.

## ETHICS STATEMENT

The Ethical Committee of Shiraz University of Medical Sciences approved this study (Code: IR.SUMS.REC.1400.524). The purpose of the study was explained to the participants, and they were assured of their anonymity and data protection. They then gave verbal informed consent. To ensure participant anonymity, questionnaires were assigned with the participants’ numbers rather than their names, and only the researchers had access to them.

## Supporting information

Supporting information.

## Data Availability

The corresponding author had full access to all of the data in this study and takes complete responsibility for the integrity of the data and the accuracy of the data analysis.
