# Supplementary material for: The prevalence and risk factors of death anxiety and fear of COVID‐19 in an Iranian community: A cross‐sectional study
Source: Health Sci Rep. 2022 Jun 19;5(4):e706. doi: 10.1002/hsr2.706 (PMC9207499; doi:10.1002/hsr2.706)
Supplement: Supplementary file 1 — Supporting information. [file HSR2-5-0-s001.docx]

**Figure S1. COVID-19 trend in Iran during the study period.**

**Figure S2. The study flowchart .**

**1,349** people approached

**982** completed interviews

**984** Interviews

**365** refused to participate

**2** didn’t complete the interviews


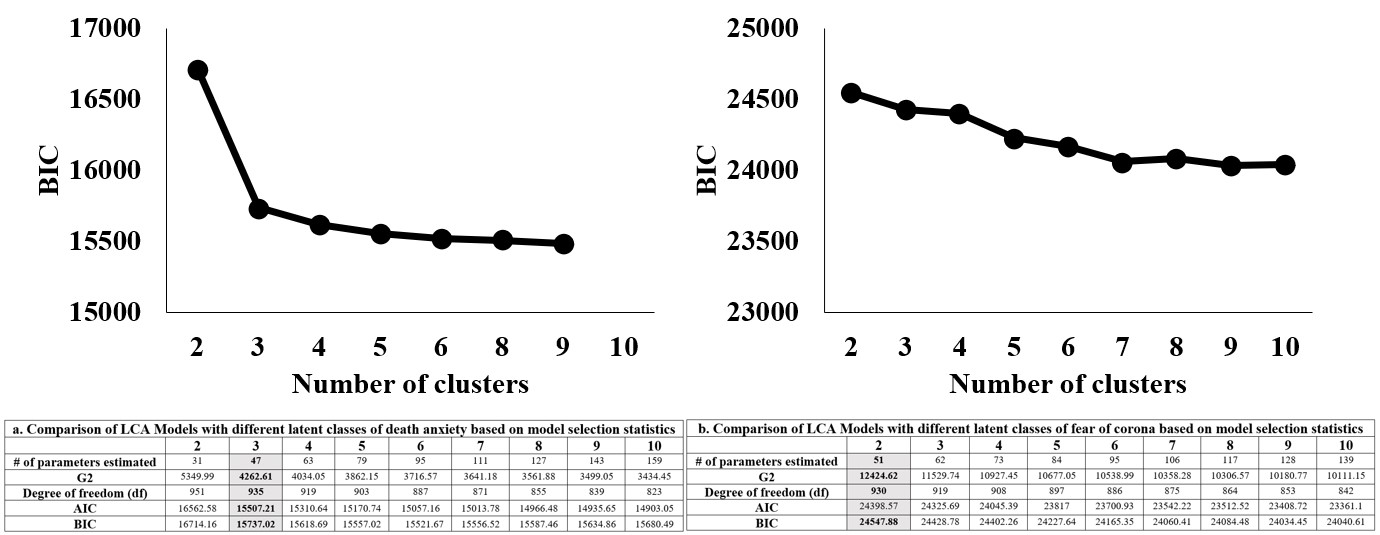


**Figure S3** comparison of latent class analysis with different latent classes of death anxiety (left) and fear of corona (right) based on model selection statistics

**Abbreviations**: BIC: Bayesian information criterion; AIC: Akaike information criterion
